# Utilization of agro-industrial by-products in *Monascus* fermentation: a review

**DOI:** 10.1186/s40643-021-00473-4

**Published:** 2021-12-16

**Authors:** Ignatius Srianta, Endang Kusdiyantini, Elok Zubaidah, Susana Ristiarini, Ira Nugerahani, Andreas Alvin, Nathania Iswanto, Bo-Bo Zhang

**Affiliations:** 1 Department of Food Technology, Faculty of Agricultural Technology, Widya Mandala Surabaya Catholic University, Jalan Dinoyo 42-44, Surabaya, 60265 Indonesia; 2 Department of Biology, Faculty of Science and Mathematic, Diponegoro University, Tembalang, Semarang, 50275 Indonesia; 3 Department of Food Science and Technology, Faculty of Agricultural Technology, Brawijaya University, Jalan Veteran, Malang, 65145 Indonesia; 4 Department of Biology, College of Science, Shantou University, 515063 Shantou, Guangdong China

**Keywords:** Agro-industry, By-product, Fermentation, *Monascus*

## Abstract

The *Monascus* fermentation industry has gained global attention. Its key products, i.e., pigments, functional food ingredients, food supplements, and medicinal use, are growing in the world’s market. Efforts to find the cost-effective substrate for *Monascus* fermentation have remained the target. This paper aimed to appraise the utilization of agro-industrial by-products (cereal, starchy tuber and root, legume, fruit, and coffee processing) as a cost-effective substrate for *Monascus* fermentation. The specific objective was to review the by-products pre-treatment, the fermentation process, product yield, and the bioactivity of the fermented products. Among all the by-products that could be used as the fermentation substrate, cereal brans do not need pre-treatment, but others need a suitable pre-treatment step, e.g., cassava peel, okara, and jackfruit seed to list a few, that need to be powdered beforehand. Other substrates, such as corn cob and durian seed, need soaking and size reduction through the pre-treatment step. During fermentation, *Monascus* produce many pigments, monacolin K, associated with rise in phenolic and flavonoid contents. These products possess antioxidant, antihypercholesterol, antidiabetes, and antiatherosclerosis activities which underpin their health significance. In conclusion, we report in this review the agro-industrial by-products which have potential prospects for pigments, functional food ingredients, food supplements, and therapeutic usages produced from *Monascus* fermentation.

## Introduction

*Monascus* fermentation has been practiced for centuries in Asian countries. In past traditional way, people were carrying out the fermentation through solid-state fermentation (SSF) using rice as substrate. The *Monascus* fermentation product is also known as ‘angkak,’ ‘anka,’ ‘red mold’ rice, ‘beni-koji,’ ‘angquac,’ or ‘red yeast’ rice. People use the *Monascus* fermentation product as a natural food colorant, food supplement, and in traditional medicine. The products have also been widely used by the community to increase thrombocytes in the blood of dengue fever patients (Srianta et al. 2014a, b; Chen et al. 2015; Prayoga and Tjiptaningrum 2016).

During the fermentation process, *Monascus* can produce various secondary metabolites, especially pigments and monacolin K. *Monascus* pigments which are categorized into three (3) groups based on the color: red, orange, and yellow. *Monascus* pigments compounds that have long been known are Monascorubramine and Rubropunctamine (red), Monascorubrin and Rubropunctatin (orange), Monascin, and Ankaflavin (yellow). By 2017, there was as many as 111 *Monascus* pigments compounds which have been identified (Chen et al. 2019). As a natural coloring, *Monascus* pigments have been widely used by the food industry for meat and fish products, rice wine, bread, biscuits, and beverage (Srianta et al. 2014a, b). Many researchers reported *Monascus* pigments compounds to have bioactivity, for example, anti-inflammatory, anticancer, antimicrobial, antiobesity, and antidiabetic (Akihisa et al. 2005; Hsu et al. 2011; Feng et al. 2012; Shi and Pan 2012; Vendruscolo et al. 2014). Meanwhile, monacolin K which is a secondary metabolite of *Monascus* has been known to have antihypercholesterolemia activity, through inhibition of hydroxymethylglutaryl-CoA (HMG-CoA) reductase activity [a key enzyme in the cholesterol biosynthesis pathway] (Lachenmeier et al. 2012). Pigments and monacolin K are produced by *Monascus* through polyketide pathways involving the polyketide synthase enzyme group (Hajjaj et al. 2000). Pigments and monacolin K production are influenced by the strain of *Monascus* fungi, the fermentation substrate, and the conditions during fermentation (Miyake et al. 2008; Feng et al. 2012; Chen et al. 2019; Kraboun et al. 2019). Moreover, *Monascus* fungi produce additional bioactive compounds, such as dimerumic acid, deffericoprogen, and γ-aminobutyric acid (GABA) which can improve the bioactivities of the substrates (Lai et al. 2019; Su et al. 2003).

Innovative efforts to find substrates other than rice for the *Monascus* fermentation have continued to evolve. Various agricultural products, namely, cereals (wheat, corn, sorghum, finger millet), tubers (cassava, sweet potato, dioscorea), and legumes (soybeans, green beans), have been used as a fermentation substrate for the fermentation process (Venkateswaran and Vijayalakshmi 2010; Srianta et al. 2014a, b; Kraboun et al. 2013; Srianta and Harijono 2015; Srianta et al. 2016). Moreover, various by-products of agro-industry have potential as a cost-effective *Monascus* fermentation substrate. Thus, this review appraises the utilization of the agro-industry by-products for the *Monascus* fermentation, including pre-treatment, fermentation process, product yield, and bioactivity of the fermented products.

## *Monascus* fungi

*Monascus* is edible fungi that belong to the *Monascaceae* family, the *Eurotiales* order, *Eurotiomycetes* class, and *Eumycota* phylum. *Monascus* has been traditionally used in Asia for centuries to produce various fermentation products. The *Monascus* mold was first isolated from angkak collected in Java by Dutch researchers. *Monascus purpureus* is the species that was first published scientifically, and subsequently other species, such as *Monascus ruber*, *Monascus pilosus*, and *Monascus sanguineus* (Lin et al. 2008; Patakova 2013; Shao et al. 2014). Species of Monascus that are often used in the fermentation are *Monascus purpureus*, *Monascus pilosus*, and *Monascus ruber.*

Monascus can grow well on various media that contain carbon sources in the form of monosaccharide, disaccharides, and starches. Monascus also can grow on pectin, cellulose, and ethanol (Patakova 2013). In its culture maintenance, *Monascus purpureus* is generally grown on Potato Dextrose Agar (PDA), Sabouraud’s Dextrose Agar (SDA), or Czapek Agar at 29–32 °C (Pattanagul et al. 2007). Propagation can be done asexually and sexually, each through the formation of conidia and ascospores. The special characteristic of *Monascus* is its ability to produce pigments so that when grown on agar slant, the mycelia that are initially white will turn yellow, orange, and then red. A high throughput screening system to determine a high pigments-producing strain of *Monascus purpureus* has been developed (Tan et al. 2014). Research on the genetic and genomic aspects of the *Monascus* has long since been carried out. However, only a few groups of researchers succeeded in sequencing the genome of the *Monascus*. Yang et al. (2015) is considered to be the first publication of the *Monascus* mold genome. The research group from China successfully sequenced the genome of *Monascus* purpureus YY-1, a strain of *Monascus* that has been used in industries that produce *Monascus* pigments. Furthermore, in 2019, a group of researchers in Japan succeeded in sequencing the genomes of *Monascus purpureus* GB-01 (a strain of *Monascus* fungi used in industries producing *Monascus* pigments) and the genome sequences were deposited on DDBJ/GenBank (Kumagai et al. 2019).

*Monascus* produces pigments and monacolin K through polyketide pathways involving the polyketide synthase (PKS) enzyme. According to Watanabe and Ebizuka (2004), PKS has 11 active side domains that are well defined, namely, starter acyltransferase, acyltransferase, acyl carrier protein, β-ketoacyl synthase, β-ketoreductase, dehydratase, enoyl reductase, thioesterase, methyltransferase, product domain templates, client cyclases, and condensation. Each domain has different functions ranging from loading unit starter acetyl-CoA and malonyl-CoA, elongation of acyl units to the polyketide condensation.

In the solid-state fermentation process, *Monascus* grow and multiply by first penetrating the substrate with its mycelium so that it could associate well with the media. This makes it difficult to monitor the growth because the mold is difficult to be separated from the substrate. Therefore, analysis of *Monascus* biomass is generally carried out through measurement of glucosamine level, which is a compound of the *Monascus* cell walls (Babitha et al. 2006). The analytical methods of *Monascus* secondary metabolites have been developed. Pigment content analysis can be carried out through absorbance measurements at the wavelengths of the yellow, orange, and red spectra. In addition, the HPLC (high-performance liquid chromatography) method with Photodiode array detector and LC–MS has been developed to determine pigments content and composition. Meanwhile, monacolin K level is determined by using the HPLC method with a UV detector (Miyake et al. 2008; Feng et al. 2012).

## Utilization of agro-industrial by-products in *Monascus* fermentation

In every process of agricultural products, some by-products are always generated. The latter are usually of low economic value, and some are even being discarded. Many types of agro-industrial by-products have the potential to be further processed into a more significant economic value products. Table 1 shows the agro-industrial by-products of cereal, tuber and root, legume, fruits, and coffee processing that have been utilized as a substrate for *Monascus* fermentation. Some agro-industrial by-products require pre-treatment and some do not, depending on the characteristics of each material.

**Table 1.** Agro-industrial by-products production, characteristics, and pre-treatment for *Monascus* fermentation

Agro-industrial by-product	Potential global production (tons/year)*	Physical and Chemical characteristics	Pre-treatment for *Monascus* fermentation
*Cereal processing*			
Corn bran	115 × 10^6^	Rich in protein (9.3 g/100 g), carbohydrate (27.6 g/ 100 g), phenolic content (68.9 mg GAE/g), and antioxidant activity (416.1 µM Trolox/g)^a^	No pre-treatment
Rice bran	78 × 10^6^	Powder, high vitamin B1 and zinc^b^ High functional compound and antioxidants^c^	No pre-treatment
Wheat bran	73 × 10^6^	Rich in nutrition, starch content (23.3%), high volumetric specific surface area, porous, good for SSF substrate^d^	No pre-treatment
Barley bran	14 × 10^6^	Poor water absorption^e^	Grinding, soaking, drying
Sorghum bran	6 × 10^6^	Starchy pericarp, high polyphenol content^f^	Soaking and sterilization
Corn cob	330 × 10^6^	Yellow to brown, 32.3–45.6% cellulose, 39.8% hemicellulose^g^	Washing, drying with direct sunlight, grinding, pressing, drying, sterilization
*Starchy tuber and root processing*			
Potato peel	55 × 10^6^	Powder, high water content, containing 7.8 g carbohydrates in starch of 100 g potato peel^h^ Contains of non-starch polysaccharides, lignin, polyphenol, protein, and less of lipid^i^	Powdering
Cassava peel	28 × 10^6^	Powder, rich in carbohydrate^j^	Powdering
Sweet potato peel	9 × 10^6^	Powder, rich in carbohydrate (65–70%)^k^	Powdering
Cassava residue	47 × 10^6^	Powder, more porous, rich in carbohydrate (660 g/kg dry basis), high fiber content^l^	Powdering
*Legume processing*			
Okara	170 × 10^3^	Poor in nitrogen and rich of fiber (50%), protein (25%), fat (10%)^m^	Drying
Soy bran	28 × 10^6^	Powder, rich in carbohydrate (9 g/kg dry basis), protein (480 g/kg dry basis), and phosphorus (7 g/kg dry basis)^l^	Powdering
*Fruit processing*			
Coconut testa	3 × 10^6^	Brown, thin: 0.2 mm thick, high antioxidant (phenolic content, tocopherol, tocotrienol), and radical scavering^c^	Powdering
Coconut residue	6 × 10^6^	Powder, rich in carbohydrate^n^	Drying, grinding
Jackfruit seed	390 × 10^3^	Particle size 0.4 and 0.6 mm, high moisture content, stable color pigments on a wide range pH, and 36.7% starch content^o^	Soaking, size reduction
Durian seed	100 × 10^3^	Brown, adhesive, firm, high moisture content (60%) and 18.92% starch content^p^	Boiling, soaking, size reduction
*Coffee processing*			
Coffee residue	15 × 10^6^	Dried coffee fermented residue, high total phenols (10.857 mg GAE g residue), bioactive compound with antioxidant action^q^	Drying

*FAOSTAT, 2020; ^a^Almeida et al., 2019; ^b^Zubaidah and Dewi, 2014; ^c^Jamaluddin et al., 2016; ^d^Manan and Webb, 2019; ^e^Wen et al., 2020; ^f^Srianta and Harijono, 2015; ^g^Velmurugan et al., 2011; ^h^Embaby et al., 2018; ^i^Sepelev and Galoburda, 2015; ^j^Fatimah et al., 2014; ^k^Sehrawat et al., 2017a, b; ^l^Carvalho et al., 2007; ^m^Colletti et al., 2020; ^n^Nimnoi and Lumyong, 2011; ^o^Babitha et al., 2006; ^p^Srianta et al., 2012; ^q^Brito et al., 2012

In the fermentation process, the moisture content of these by-products may need to be adjusted to facilitate optimum growth and metabolism of *Monascus*. Nutrients, such as nitrogen sources and minerals, may be required, followed by sterilization step at 121 °C for 15–20 min. After cooling to a temperature suited to *Monascus growth*, the substrate is then inoculated with a *Monascus* starter culture. The cultures used in various literature include *Monascus purpureus, Monascus pilosus,* and *Monascus ruber*. After this step, incubation is conducted at its optimal conditions in 7–14 days following the fermentation process, the resulting product is dried at 45–55 °C. Table 2 summarizes the SSF process of agro-industrial by-product substrate.

**Table 2:** Summary of *Monascus* SSF process on agro-industrial by-product substrate

Agro-industrial by-product	SSF culture and condition	Product	References
Corn bran	*M*. *purpureus* ATCC 36,928; 32 °C, 16 days	Fermented corn bran with red color and important nutritional value	Almeida et al. (2019)
Rice bran	*M*. *pilosus* KCCM 60,084; 25 °C, 10 days *M*. *purpureus*; 32 °C, 12 days	Fermented rice bran with high monacolin K content. Phenolic and flavonoid contents; antioxidant activity enhanced	Cheng et al. (2016) Jamaludin et al. (2014); Razak et al. (2015)
Wheat bran	*M*. *purpureus* LPB 97; 30 °C, 7 days *M*. *purpureus* ATCC 16,436; 30 °C, 23 days	Red pigments	Babitha et al. (2007); Mousa et al. (2018)
Barley bran	*M*. *purpureus* CICC 5046; 28–32 °C, 12 days	*Monascus* fermented barley bran–coix seed with enhanced monacolin K, pigments and soluble polyphenol contents; barley bran–adlay with hypolipidemic activity	Li-Ning et al. (2017); Ding et al. (2017)
Sorghum bran	*M*. *purpureus* M9; 30 °C, 14 days	*Monascus* fermented sorghum bran containing pigments, monacolin K, and antioxidants activity	Srianta and Harijono (2015)
Corn cob	*M*. *purpureus* KACC 42,430; 30 °C, 7 days *M*. *purpureus* ATCC 16,436; 30 °C, 10 days	Pigments; stable in acidic pH, high temperature and salt solution Orange and red pigments	Velmurugan et al. (2011)
Potato peel	*M*. *sanguineus*; 28 °C, 20 days M. *purpureus* ATCC 16,436; 30 °C, 23 days	Red pigments Red pigments with antifungal activity	Padmavathi and Prabhudessai (2013); Mousa et al. (2018)
Cassava peel	*M*. *purpureus*; 30–31 °C, 14 days	Yellow, orange and red pigments	Afiandiningsih (2013)
Sweet potato peel	*M*. *purpureus* MTCC 369; 30 °C, 15 days	Yellow, orange and red pigments	Sehrawat et al. (2017a, b)
Cassava residue	*Monascus sp*., LPB -31	Yellow and red pigments	Carvalho et al. (2007)
Okara	*Monascus purpureus*	Red and orange pigments	Sun et al. (2020)
Soy bran	*Monascus purpureus*	Pigments production	Carvalho et al. (2007)
Coconut testa	*Monascus purpureus*	*Monascus* fermented coconut testa enhanced total phenolics, antioxidant potential, and radical scavenging activity compared to unfermented coconut testa	Jamaluddin et al. (2016)
Coconut residue	*M*. *purpureus* MTCC 410 and *M*. *sanguineus*	Red pigments and yellowish-orange pigments	Padmavathi and Prabhudessai (2013)
Jackfruit seed	*M*. *purpureus* LPB 97; 30 °C, 7 days	Red and yellow pigments	Babitha et al. (2006)
Durian seed	*M*. *purpureus*; 30 °C, 14 days	Durian seed as optimum substrate for *Monascus sp*. KJR2 to produce pigments with 50 mg/kg monacolin K	Srianta et al. (2012)
Coffee residue	*Monascus purpureus*	*Monascus* fermented coffee residues high in polyphenol and bioactive compound with antioxidant action that have beneficial effect on cardiovascular disease	Brito et al. (2012)

## Cereal processing by-products

Globally, there are five (5) major kinds of cereal produced, these are corn, wheat, rice, barley, and sorghum. In the cereal milling process, bran that makes up approximately 10% of the grain is produced as a by-product. Based on the cereal production data, the annual global production potential for rice, barley, wheat, sorghum, and corn brans are 48, 14, 9.5, 6, and 100 million tons, respectively (Alauddin et al. 2017; Chakraborty and Budhwar 2019; Statista 2020). Cereal bran is generally light brown powder. It consists of aleurone layer, pericarp layers (pericarp and testa), germ, and a small portion of endosperm, which is rich in carbohydrate, protein, lipids, vitamins, minerals, and phytochemicals. When it comes to the processing of corn, corn cob which constitutes about 30% of the whole corn is largely produced. Corn cob contains polysaccharides, mainly cellulose and hemicellulose (Velmurugan et al. 2011). These by-products are underutilized resource in most developed countries and are usually used for animal feed (Srianta and Harijono 2015). These facts encourage researchers to explore potential utilization to increase their economic value.

**Fig. 1:**
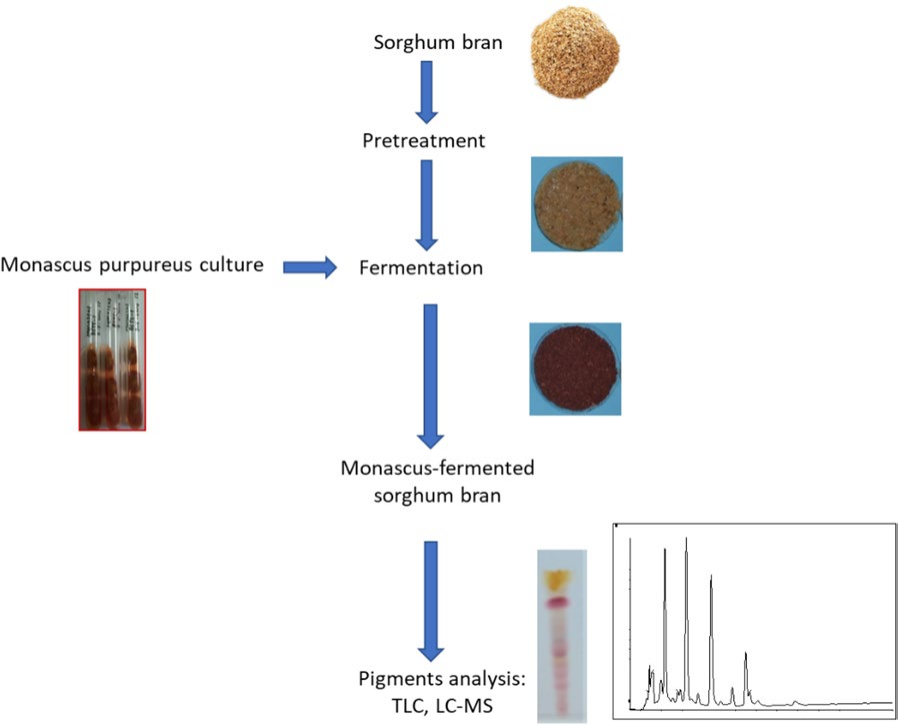
Solid-state fermentation of sorghum bran with *Monascus purpureus* for pigments production

When used as a *Monascus* fermentation substrate, the moisture content of the cereal bran needs to be adjusted to facilitate the growth and metabolism of *Monascus*. Without the addition of water, the growth and the pigments production of *Monascus purpureus* is very limited. Water and cereal bran ratio of 1:1 creates a substrate condition suitable for *Monascus purpureus* growth and pigments production. After fermentation process at 30 °C for 7 days on wheat bran and 14 days on sorghum bran (Fig. 1), the red pigments production reaches score of 3.525 and 22.90 AU/g, respectively. However, the redness value (b*) of corn bran inoculated with *Monascus* increases from 6.9 on the 4th day of fermentation to 10.2 on the 16th day fermentation (Babitha et al. 2007; Srianta and Harijono 2015; Almeida et al. 2019). Similarly, the addition of water on rice bran for the *Monascus* fermentation has also been reported by other researchers (Razak et al. 2015; Cheng et al. 2016). Cheng et al. (2016) reported that *Monascus pilosus* growth and metabolism were affected by the moisture content, which adjusted in the range of 35–50%. It was concluded that 45% is the optimum moisture content for *Monascus pilosus* KCCM60084 to grow and produce monacolin K. During the fermentation process at 25 °C for 10 days, the *Monascus pilosus* culture produces monacolin K in a considerable level (2881 mg/g dry weight), higher than that on the best-known substrate, i.e., yam (2584 mg/g dry weight). Moreover, the *Monascus* fermentation process increased the level of flavonoid and total phenolic content in rice bran substrate. These results indicate that the *Monascus purpureus* can produce β-glucosidase, which hydrolyze the conjugated phenolic compounds into free phenol. Consequently, the fermented rice bran products showed higher antioxidant activity than that of unfermented rice bran, which was evaluated by in vitro ABTS, FRAP (Ferric Reducing Antioxidant Power) and Fe chelating methods (Jamaluddin et al. 2014, 2016; Razak et al. 2015; Cheng et al. 2016). Other researchers reported that the fermented sorghum and corn brans possess DPPH scavenging activity of 7.73 and 364.82 µmol Trolox Equivalent/g, respectively (Srianta et al. 2017; Almeida et al. 2019). These findings suggested that cereal bran solely without any nutrient supplementation is a considered potential substrate to produce pigments, monacolin K, and functional food ingredients through solid-state fermentation with *Monascus* fungi.

Some researchers utilized cereal bran in a mixed substrate to improve the *Monascus* fermentation. Zubaidah and Dewi (2014) reported the effect of rice bran supplementation into the rice substrate fermented by *M*. *purpureus* on pigments and lovastatin production. Up to 10% level, the supplementation enhanced the red pigments production compared with control (0% rice bran). This is due to the rice bran enriches micronutrients, i.e., minerals, amino acids, and vitamin B1, which are essential in the polyketide pathway. At the optimum level (5% rice bran), the fermented product produces red pigments and lovastatin levels of 3.574 AU/g and 102.040 ppm, respectively. Other researchers reported *Monascus* fermentation on a combined substrate of rice bran and coconut testa and found that is better than that on coconut testa solely. The total phenolic content and antioxidant activity of fermented-mix substrate were higher than these of fermented coconut testa and fermented rice bran (Jamaluddin et al. 2016). Other reports on the utilization of barley bran in a mixed substrate with adlay in *Monascus* fermentation (Ding et al. 2017). During fermentation on adlay and barley bran substrate, the pigments, monacolin K, and soluble polyphenol production were increased. However, granulated teas made from the fermented adlay–barley bran in combination with lotus leaves have hypolipidemic activity in Sprague–Dawley rats fed a high-fat diet.

In comparison to the cereal bran, corn cob has different physical and chemical characteristics. Corn cob has a much larger size than cereal bran and higher moisture content. According to Velmurugan et al. (2011), corn cob was washed thoroughly and dried, then ground to 2 mm particle size. The prepared material was soaked in deionized water at 80 °C for 12–48 h to increase porosity and bulk density. In its utilization, therefore, it needs pre-treatment to create a suitable characteristic as a *Monascus* fermentation substrate. The pre-treatments are washing, grinding, soaking, drying, and grinding into 2 mm size particle to increase the surface area. The ground corn cob is then soaked in hot water (80 °C) for 12–48 h to increase porosity and bulk density. After soaking, it is then pressed and dried. Since the nutrients are very limited, several nutrients are supplemented into the corn cob, e.g., KH_2_PO_4_, NH_4_NO_3_, NaCl, and MgSO_4_. Water is also added to adjust the moisture content. On corn cobs substrate and under the optimum conditions, e.g., 50% of moisture content, pH 5, and 4% starter culture concentration at 30 °C for 7 days, *Monascus purpureus* KACC 42430 produces red pigments of 25.42 AU/g of a dry fermented substrate (Velmurugan et al. 2011). Another research group reported that corn cob supplementation to a medium as co-solid-state fermentation and carbon source in *Monascus* fermentation. The supplementation successfully provoked high levels of orange and red pigments production by *Monascus purpureus* ATCC 16436. Corn cob is very economical for *Monascus* pigments production.

## Tuber and root processing by-products

Tuber and root are the second in importance to cereal as global sources of carbohydrate. Potato, cassava, sweet potato, and dioscorea are the biggest tuber commodities produced. According to data from Food and Agriculture Organization, the annual production of potatoes is over 300 million tons annually.

### Potato peel

Consumption of processed potatoes is on the rise not only in the developed countries but also in developing ones. During processing of potato, potato’s peel is the major by-product generated. The losses depending on the peeling method used ranges from 15 to 40% of the first product mass (Sepelev and Galoburda 2015). Industrial processing generates up to 140 thousand tons of peels worldwide annually. Potato peel is of a zero value as by-product, which resulted in huge amounts after processing. Potato peel as a by-product of the food processing industry poses to be a totally inexpensive, valuable, and affordable raw material for the production of economically important added-value products. Traditionally potato peel waste is used for producing low-value animal feed, fertilizer, or used as the raw material of biogas.

According to the study by Padmavathi and Prabhudessai (2013), potato peel was recognized as the best substrate for *M*. *sanguineus* fermentation. Their research used SSF, three substrates, viz, orange peel, potato peel, and coconut cake. 5 g of the substrates along with distilled water was placed in a 100 ml conical flask. The pH of the medium was adjusted to 6. It was then autoclaved for 20 min at 121 °C. These substrates were inoculated with 10% of the seed culture from both strains separately and incubated with 56–60% relative humidity at 28 °C for 20 days. It was found that both strains *Monascus sanguineus* and *Monascus purpureus* MTCC 410 grew on all experimented substrates although pigments yield varied. Based on Padmavathi and Prabhudessai (2013), for *M*. *purpureus*, coconut cake with 0.73 AU/g at 510 nm showed maximum pigments yield followed by orange peel with pigments yield of 0.65 AU/g at 510 nm. For *Monascus sanguineus*, potato peel with 0.68 AU/g at 510 nm showed maximum pigments yield followed by coconut cake with 0.56 AU/g at 510 nm. SSF possesses several biological advantages when compared with submerged fermentation. Such advantages were represented as higher fermentation productivity, less catabolic repression, lower water demand, and hence, lower sterility demand due to the low water activity, cultivation of microorganisms requiring solid support, and mixed cultivation of *Monascus*.

Other study outlined that *Monascus purpureus* produce pigments on potato peel substrate with a yield of 2.636 ± 0.04 AU/g dry substrate while on rice substrate of 3.627 ± 0.03 AU/g dry substrate (Mousa et al. 2018). It showed that rice is the most suitable substrate, but potato peel is also good for red pigments production by *Monascus purpureus*. Production of maximum pigments yield by the experimental fungal strain was achieved using potato peel as a solid substrate incubated for 16 days. In the literature, the optimum incubation time for maximum pigments production varies from one strain to another. Ahmad and Panda (2011) used 14 days of incubation for pigments production. A shorter incubation period (7 days) was reported for pigments production by some other authors (Babitha et al. 2006; Rajeswari et al. 2014). Maximum pigments production by the experimental fungal strain was achieved by potato peel substrate with a moisture content of 75%. In partial accordance with these results, Ahmad and Panda (2014) used a rice-based medium with a moisture content of 70%. Some authors used 56–60% moisture content for maximum pigments production (Dikshit and Tallapragada 2011; Padmavathi and Prabhudessai 2013).

### Sweet potato peel

Maloney et al. (2014) reported the findings of the analysis of the proximate composition of sweet potato peel, i.e., moisture 4.74–4.76%, carbohydrates 77.0–76.4%, protein 6.40–6.49%, fat 2.33–2, 65%, and ash 9.47–9.70%. Sweet potato peels were suspected to be *Monascus* substrate based on their nutritional value. Sweet potato peels were washed, dried, crushed, sieved, added nutrients, adjusted the moisture content, sterilized, and inoculated with *Monascus* culture. After that it is fermented and dried.

The main objective of Sehrawat et al. (2017a), Sehrawat et al. (2017b) research was to optimize the media and process parameters for bio-pigments extraction with *Monascus purpureus* MTCC 369. The pigments of *Monascus purpureus* MTCC 369 are natural source of colorant. Bio-pigments production was carried out using solid-state fermentation. Sweet potato peel powder and pea pod powder were used as *Monascus purpureus* substrates, fermented at optimized condition 32 °C for 8 days 9 h and pH 5.4. There was an increase in pigments production up to 7.81% (w/w) sweet potato peel powder and up to 3.93% (w/w), respectively, with a final yield of 21 CVU/g. The decrease in bio-pigments production may be caused by C/N ratio. Moreover, increasing temperature over 32 °C may also decrease the bio-pigments production.

### Cassava peel

Cassava peel has been unsuitable for animal feed since its high content of cyanogenic glucosides. Okpako et al. (2008) reported the proximate composition of cassava peel in terms of moisture (8.60%), carbohydrate (64.51%), protein (10.60%), fat (3.52%), and ash (6.54%). However, the cassava pulp contained different values for moisture (3.60%), carbohydrates (72.72%), protein (0.93%), fat (1.63%), and ash (1.52%) (Enenebeaku et al. 2016). Based on their chemical composition, these by-products have the potential as fermentation substrates for *Monascus* fermentation.

Utilization of these ingredients in the development of *Monascus* fermentation products was carried out through the process of washing and drying the inner cassava peel, crushing, and sieving. Nitrogen source and mineral were added to the cassava peel flour, the moisture content was adjusted, then sterilized, and inoculated with *Monascus* culture. Afterward, the inoculated substrate was fermented and dried.

Afiandiningsih (2013) reported that cassava peel substrate with a starter culture concentration of 10% produced the highest level of *Monascus* pigments. The resulting product contained yellow, orange, and red pigments of 1.63, 0.96, and 1.09 AU/g, respectively. Based on Fatimah et al. (2014), cassava peels flour with the addition of 10% rice bran showed the highest red pigments production (5.6 CVU/gds) and 47% of water content. The results showed that the addition of rice bran to cassava peel substrate could increase *Monascus* red pigments production. On cassava bagasse substrate, fermentation products contain red pigments of 15.7 AU/g. Adjustment of moisture content at 70% can increase pigments production up to 25 AU/g (Carvalho et al. 2007).

## Legume processing by-products

Legumes are agricultural products that contribute the largest source of vegetable protein, especially soybeans. The removal of the husk is usually needed for the processing of soybeans so that the skin of the soybean (soybean bran) is collected as a by-product. However, in the processing of soybeans into soy milk and tofu, other by-products are produced in large quantities in the form of soybean residue or okara. Soybean bran contains 40.0% carbohydrates and 48.0% protein (Carvalho et al. 2007), while okara has a proximate composition of carbohydrate (3.8–5.3%), fiber (52.8–58.1%), protein (25.4–28.4%), fat (9.3–10.9%), and ash (3.0–3.7%) (Li et al. 2012).

In the utilization of soybean bran, a pre-treatment process of drying, grinding, and sifting to obtain soybean bran with a size of 0.8–2.0 mm is needed (Carvalho et al. 2007). If Okara is used, preliminary treatment is carried out in the form of drying (Japakaset et al. 2009) or drying and grinding (Nimnoi and Lumyong 2011). In the research of Nimnoi and Lumyong (2011), the addition of nitrogen and mineral sources was carried out. The next stage is the same for both soybean bran and okara, which is to adjust water content, sterilization, cooling, inoculation with *Monascus* starter culture, fermentation, and drying the product.

Red pigments produced by *Monascus purpureus* on soybean bran substrate can reach up to 22 AU/g (Carvalho et al. 2007). The red pigments were measured by a spectrophotometer at 500 nm. In okara substrate, *Monascus purpureus* growth reached its maximum on the 7th day, after which it decreased (Japakaset et al. 2009). Red pigments production by *Monascus purpureus* is relatively low at around 3 AU/g (Nimnoi and Lumyong 2011). The low production of red pigments might be due to the limited carbon source in the substrate. This is proven by the addition of carbon sources in the form of galactose, glucose, mannitol, psicose, sorbose, and xylitol at levels 4 and 8% to increase the production of red pigments. The highest increase occurred in the addition of glucose, where the production of red pigments reached around 23 AU/g. Japakaset et al. (2009) reported that *Monascus purpureus* produces monacolin K. At its optimum conditions, i.e., pH 4, 30 °C, and 25% water content, monacolin K production reaches 109.23 mg/kg. Monacolin K levels in rice substrate were 481 mg/kg. *Monascus* fermentation products from soybean bran and okara have not been tested for their bioactivity yet.

## Fruit processing by-products

Fruits are agricultural commodities and a source of fiber, vitamins, and minerals. The by-product of fruit processing varies. Some of the by-products used as a *Monascus* fermentation media are coconut residue, jackfruit seeds, and durian seeds. Moorthy and Viswanathan (2009) reported the results of the analysis of coconut dregs, namely, water content 9.54%, protein 22.75%, fat 2.89%, crude fiber 12.11%, and ash content 7.41%. Jackfruit seeds contain 15.88% water, 71.46% carbohydrates, 5.78% protein, 1.77% fat, and 2.62% ash (Islam et al. 2015). Durian seeds contain 51.5% water, 43.6% carbohydrates, and 2.6% protein (Brown, 1997).

**Fig. 2:**
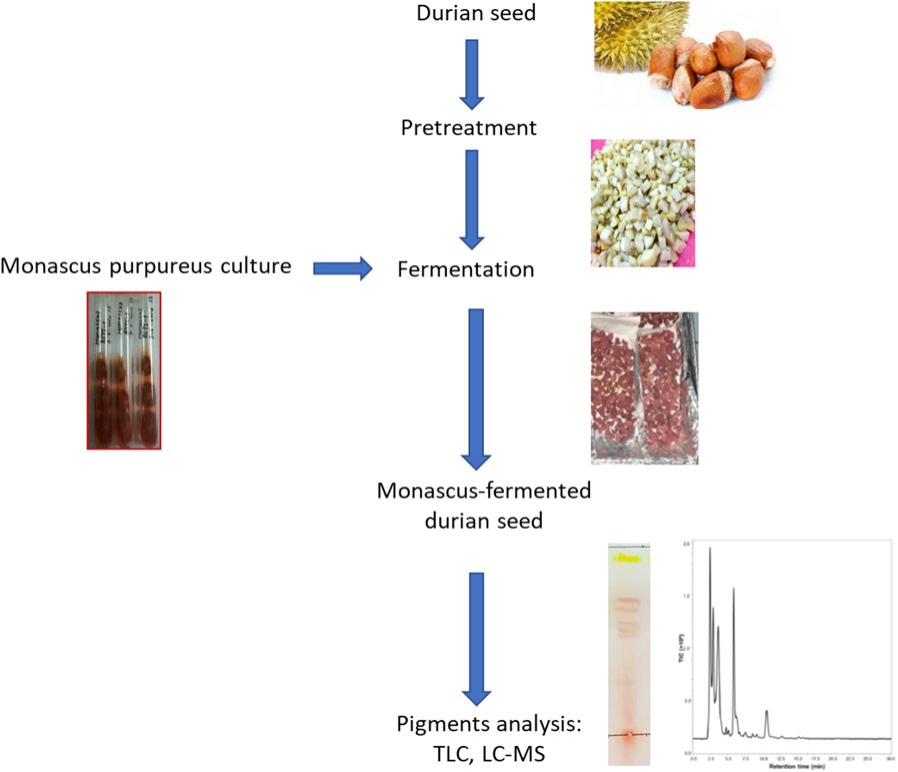
Solid-state fermentation of durian seed with *Monascus purpureus* for pigments production

Jackfruit seeds need to be dried and ground before being used as a medium for *Monascus* fermentation. When using durian seeds, it is necessary to soak them in a lime solution, peel them, cut them into small sizes, and adjust their water content (Fig. 2). Following the adjustment of water content, the next process steps were the same as the general process, namely, sterilization, cooling, inoculation, incubation, and product drying (Babitha et al. 2006; Nimnoi and Lumyong 2011; Srianta et al. 2012).

According to the research by Nimnoi and Lumyong (2011), using coconut residue as the media for fermentation, *Monascus purpureus* produced red pigments at a very low level of 0.59 AU/g. Their experiment has shown that the addition of carbon sources in the form of galactose, glucose, mannitol, psicose, sorbose, and xylitol can increase the production of red pigments with different levels. The addition of 8% glucose results in increased production of red pigments to about 65 AU/g (Nimnoi and Lumyong 2011). In jackfruit seed flour substrate, *Monascus purpureus* can grow well and produce red and yellow pigments of 19.5 and 19.0 AU/g, respectively. Supplementation of several types of carbon sources, such as rice flour, tapioca, sucrose, sorbitol, xylose, and lactose, does not increase the pigments production; significantly, it can even decrease the yield of pigments. Meanwhile, supplementation of nitrogen sources in the form of monosodium glutamate, peptone, okara, and chitin by 1% can increase the production of pigments. Monosodium glutamate is a nitrogen source which can provide the highest increase in the production of red and yellow pigments reaching 30.8 and 25.5 Au/g, respectively (Babitha et al. 2006). In durian seed substrate, *Monascus* can grow well and produce pigments and monacolin K. Production of water-soluble yellow, orange, and red pigments were 11.17, 8.52, and 8.11 AU/g, while ethanol-soluble pigments were 3.86, 2.51, and 3.73 AU/g. However, monacolin K production was 50 mg/kg (Srianta et al. 2012). The bioactivity of the *Monascus* fermented products with coconut residue and jackfruit seeds have not been tested, while *Monascus* fermented durian seed product has been tested for in vitro antioxidant and antidiabetic activities, and in vivo antihypercholesterol and antidiabetic activity. The antioxidant activity of the fermented product was tested by DPPH (2,2-diphenyl-1-picrylhydrazyl), FRAP, and phosphomolybdenum methods by inhibiting 56.26%, 93.45 mg GAE/g, and 256 mg GAE/g, respectively (Srianta et al. 2014a, b). Additionally, the ethanol extract of durian seed fermentation products has inhibitory activity against the α-glucosidase enzyme with IC_50_ of 70.7 µg/mL (Srianta et al. 2013). Nugerahani et al. (2017) reported that administration of 0.15 g of *Monascus* fermented durian seeds extracted with water was able to reduce glucose and cholesterol levels in the blood of Wistar rats by 12.89 and 49.30%, respectively.

Screening of substrates for GABA synthesis was carried out using different agro-industrial residues, e.g., wheat bran, tamarind seed, coconut oil cake, and jackfruit seed. Five grams of each substrate was taken separately and placed in a 250 ml conical flask to which 30 ml of basal medium was added. The basal medium comprised 100 g dextrose, 2 g KNO_3_, 10 g peptone, 2 g NH_4_ H_2_PO_4_, 0.5 g MgSO_4_∙7H_2_O, and 0.1 g CaCl_2_∙2H_2_O in 1000 ml distilled water. The medium was adjusted to pH 6.0 (Dikshit and Tallapragada 2011). The use of synthetic media on an industrial scale for the production of bioactive compounds from microbial sources was not economical as far as the cost is concerned. Therefore, the development of low-cost processes is necessary. Keeping this in mind, various agro-waste residues were screened; among these, coconut oil cake gave the maximum yield (7.74 mg/gds), followed by jackfruit seed (6.96 mg/gds) and wheat bran (6 mg/gds), while the yield obtained from tamarind seeds was low (2.12 mg/gds). Yield is a metric that results from dividing the amount of pigments or other metabolites produced divided by the total amount of substrate in the fermentation. In the present work, an extremely economical agricultural residue, i.e., coconut oil cake, was used. From the model developed in that study, the optimum GABA yield was estimated to be 15.53 mg/gds with an added MSG concentration of 0.05 g, a pH of 7.5, and an incubation period of 20 days. To validate the results predicted by the model, a test was run under these conditions and the GABA yield was found to be 15.31 mg/gds, which was close to the predicted yield. This substantiated the model. Comparing the GABA yield per unit substrate invested in terms of monetary as well as utility value, the results of the presented study were encouraging. This can be deemed a good use of coconut oil cake, which is produced in large amounts and may otherwise go to waste.

## Coffee processing by-product

In various countries, the coffee business is currently on the rise. This phenomenon had a great impact on the rise of the coffee processing business. By-products in the form of coffee residue will be generated after brewing coffee. Aguilar-Raymundo et al. (2019) reported the proximate composition of a dry coffee residue, i.e., moisture 6.0%, fat 12.4%, protein 8.2%, ash 1.5%, and carbohydrate 38.1%.

Before it is used in the fermentation process, the coffee residue is dried to a water content of around 10%. Nitrogen sources and several types of minerals were added to the dried coffee residue, and the moisture content was adjusted. After that, it was inoculated with *Monascus* ruber culture, then incubated at 28 °C for 13 days. The fermentation product was then dried. Brito et al. (2012) reported that *Monascus* fermented coffee residue has a total phenol level of 10,867 mg GAE/g, higher than that of unfermented coffee residue (7772 mg GAE/g). The product was tested for its in vivo antiatherosclerosis activity using Apo E. mice. The test results indicated that the addition of 2% of the fermented coffee residue can reduce the lesion area by 26.4%. The results of this study indicated that coffee residue fermentation products have a positive effect in reducing the formation of atheroma plaque.

When using agro-industrial by-products for the Monascus fermentation, one of the major problems is the relatively low productivity of Monascus metabolites. In general, the pigments production (AU/g) and monacolin K production (mg/kg) were lower than that achieved by using normal cereals as substrate. Several strategies to increase the production of pigments and monacolin K are combining 2 complementary by-products, such as corn cob and glycerol, durian seeds and molasses, and durian seeds and okara, adding carbon source, nitrogen source, and minerals at optimal levels.

## Conclusions

There are a lot of agro-industrial by-products in global food production which vary from cereal’s bran, peels from tubers, and fruit’s seeds which are bio-degradable to waste from industry, which still contain a lot of benefits. It is therefore necessary to utilize these wastes, one of these possible utilizations is to be act as substrate for *Monascus* fermentation.

In this review, many bio-degradable wastes are very promising and showed great potential for applications in health and or to be used in the development of *Monascus* fermentation. Some of these wastes produce biochemicals that are beneficial to our health. Fermented rice bran, okara, durian seeds, and coffee grounds have important bioactivity for health, namely, antioxidants, antihypercholesterol, antidiabetic, and antiatherosclerosis. Therefore, further studies are warranted regarding the application of various fermented products, both as natural colorant and functional food ingredients.

## Data Availability

Not applicable.

## Abbreviations

AU: Absorbance unit
CVU: Color value unit
DPPH: 2,2-Diphenyl-1-picrylhydrazyl
FRAP: Ferric reducing antioxidant power
GABA: γ-Aminobutyric acid
GAE: Gallic acid equivalent
HMG-CoA: Hydroxymethylglutaryl-CoA
HPLC: High-performance liquid chromatography
PDA: Potato dextrose agar
SDA: Sabouraud’s dextrose agar
SSF: Solid-state fermentation

## References

[CR1] Afiandiningsih D (2013) Pengaruh Konsentrasi Inokulum (*Monascus purpureus*) Terhadap Produksi Pigmen pada Substrat Tepung Kulit Singkong (*Manihot esculenta*) (Effect of inoculum concentration of *Monascus purpureus* to the production of pigments in the cassava peels flour (*Manihot esculenta*)). http://repository.upi.edu/id/eprint/4159. Accessed 21 June 2020

[CR2] Aguilar-Raymundo VG, Sanchez-Paez R, Gutierrez-Salomon AL, Barajas-Ramirez JA (2019). Spent coffee grounds cookies: sensory and texture characteristics, proximate composition, antioxidant activity, and total phenolic content. J Food Process Preserv.

[CR3] Ahmad M, Panda BP (2011). Screening of nutrient parameters for red pigment production by *Monascus purpureus* MTCC 369 under solid state fermentation by using placket burman experimental design. Int J Pharm Front Res.

[CR4] Akihisa T, Tokuda H, Ukiya M, Kiyota A, Yasukawa K, Sakamoto N, Kimura Y, Suzuki T, Takayasu J, Nishino H (2005). Anti-tumor initiating effects of monascin, an azaphilonoid pigment from the extract of *Monascus pilosus* fermented rice (red-mold rice). Chem Biodivers.

[CR5] Alauddin Md, Islam J, Shirakawa H, Koseki T, Ardiansyah MK, Waisundara V, Shiomi N (2017). Rice bran as a functional food: an overview of the conversion of rice bran into a superfood/functional food. Superfood and functional food—an overview of their processing and utilization.

[CR6] Almeida ABD, Lima TMD, Santos NH, Santana RV, Santos SCD, Egea MB (2019). An alternative for corn bran byproduct: fermentation using *M*. *purpureus*. Nutr Food Sci.

[CR7] Babitha S, Soccol CR, Pandey A (2006). Jackfruit seed—a novel substrate for the production of *Monascus* pigments through solid-state fermentation. Food Technol Biotechnol.

[CR8] Babitha S, Soccol CR, Pandey A (2007). Solid-state fermentation for the production of *Monascus* pigments from jackfruit seed. Bioresour Tech.

[CR9] Brito LF, de Queirós LD, Peluzio MCG, Ribeiro SMR, da Matta SLP, de Queiroz JH (2012). Effect of dry coffee residues fermented with *Monascus ruber* on the metabolism of Apo E mice. Arg Bras Cardiol.

[CR10] Brown MJ (1997) Durio—a bibliographic review. In: Arora RK, Rao VR, Rao AN (eds) New Delhi, India

[CR11] Carvalho JC, Oishi BO, Woiciechowski AL, Pandey A, Babitha S, Soccol CR (2006). Effect of substrates on the production of *Monascus* biopigments by solid-state fermentation and pigment extraction using different solvents. Indian J Biotechnol.

[CR12] Carvalho AA, Lovatto PA, Hauschild L, Andretta I, Lehnen CR, Zanella I (2007). Processing of full-fat soybean and the use in diets for pigs: digestibility and metabolism. Rev Bras Zootecnia.

[CR13] Chakraborty M, Budhwar S (2019). Critical analysis of wheat bran as therapeutic source. Int J Trend Sci Res Dev.

[CR14] Chen W, He Y, Zhou Y, Shao Y, Feng Y, Li M, Chen F (2015). Edible filamentous fungi from the species *Monascus*: early traditional fermentations, modern molecular biology, and future genomics. Compr Rev Food Sci Food Saf.

[CR15] Chen W, Feng Y, Molnar I, Chen F (2019). Nature and nurture: confluence of pathway determinism with metabolic and chemical serendipity diversifies *Monascus azaphilone* pigments. Nat Prod Rep.

[CR16] Cheng J, Choi B, Yang S, Suh JW (2016). Effect of fermentation on the antioxidant activity of rice bran by *Monascus pilosus* KCCM60084. J Appl Biol Chem.

[CR17] Colletti A, Attrovio A, Boffa L, Mantegna S, Cravotto G (2020). Valorisation of by-products from soybean (*Glycine max* (L.) Merr.) processing. Molecules.

[CR18] Dikshit R, Tallapragada P (2011). *Monascus purpureus*: a potential source for natural pigment production. J Microbiol Biotechnol Res.

[CR19] Ding Y, Pu L, Kan J (2017). Hypolipidemic effects of lipid-lowering granulated tea preparation from *Monascus*-fermented grains (adlay and barley bran) mixed with lotus leaves on Sprague–Dawley rats fed a high-fat diet. J Funct Foods.

[CR20] Embaby AM, Hussein MN, Hussein A (2018). *Monascus* orange and red pigments production by *Monascus purpureus* ATCC16436 through co-solid state fermentation of corn cob and glycerol: an eco-friendly environmental low cost approach. PLoS ONE.

[CR21] Enenebeaku CK, Enenebeaku UE, Ezejiofor TIN (2016). Proximate composition and production of bioethanol from cassava bagasse using hydrochloric acid and *Saccharomyces cerevisiae* (Baker’s yeast). J Biol Chem Res.

[CR22] FAOSTAT (2020) Crops. http://www.fao.org/faostat/en/#data/QC. Accesed 21 June 2020

[CR23] Fatimah S, Suprihadi A, Kusdiyantini E (2014). Produksi dan kestabilan pigmen merah kapang *Monascus* sp. menggunakan media tepung kulit singkong dengan penambahan bekatul pada konsentrasi yang berbeda (Production and stability of red pigment fungus *Monascus sp*. using cassava peel flour media with the addition of bran at different concentrations). J Biol.

[CR24] Feng Y, Shao Y, Chen F (2012). *Monascus* pigments. Appl Microbiol Biotechnol.

[CR25] Hajjaj H, Francois JM, Goma G, Blanc PJ (2000). Effect of amino acids on red pigments and citrinin production in *Monascus ruber*. J Food Sci.

[CR26] Hsu YW, Hsu LC, Liang YH, Kuo YH, Pan TM (2011). New bioactive orange pigments with yellow fluorescence from *Monascus*-fermented dioscorea. J Agric Food Chem.

[CR27] Islam MS, Begum R, Khatun M, Dey KC (2015). A Study on nutritional and functional properties analysis of jackfruit seed flour and value addition to biscuits. IJERT.

[CR28] Jamaluddin A, Rashid NYA, Razak DLA, Sharifudin SA, Long K (2014). Effect of fungal fermentation on tyrosinase and elastase inhibition activity in rice bran. Agric Agric Sci Procedia.

[CR29] Jamaluddin A, Razak DLA, Rashid NYA, Sharifudin SA, Kahar AA, Saad AZM, Long K (2016). Effects of solid state fermentation by *Monascus purpureus* on phenolic content and biological activities of coconut testa and rice bran. J Teknol.

[CR30] Japakaset J, Wongkhalaung C, Leelawatcharamas V (2009). Utilization of soybean residue to produce monacolin K-cholesterol lowering agent. Songklanakarin J Sci Technol.

[CR31] Kraboun K, Tochampa W, Chatdamrong W, Kongbangkerd T (2013). Effect of monosodium glutamate and peptone on antioxidant activity of monascal waxy corn. Int Food Res J.

[CR32] Kraboun K, Kongbangkerd T, Rojsuntornkitti K, Phanumong P (2019). Factors and advances on fermentation of *Monascus* sp. for pigments and monacolin K production: a review. Int Food Res J.

[CR33] Kumagai T, Tsukahara M, Katayama N, Yoai K, Aburatani S, Ohdan K, Fujimori KE (2019). Whole-genome sequence of *Monascus purpureus* GB-01, an industrial strain for food colorant production. Microbiol Resour Announc.

[CR34] Lachenmeier DW, Monakhova YB, Kuballa T, Löbell-Behrends S, Maixner S, Kohl-Himmelseher M, Waldner A, Steffen C (2012). NMR evaluation of total statin content and HMG-CoA reductase inhibition in red yeast rice (*Monascus* spp.) food supplements. Chin Med.

[CR35] Lai J, Ke B, Hsu Y, Lee C (2019). Dimerumic acid and deferricoprogen produced by *Monascus purpureus* attenuate liquid ethanol diet-induced alcoholic hepatitis via suppressing NFκB inflammation signalling pathways and stimulation of AMPK-mediated lipid metabolism. J Funct Foods.

[CR36] Lee JE, Vadlani PV, Faubion J (2017). Corn bran bioprocessing: development of an integrated process for microbial lipids production. Bioresour Technol.

[CR37] Li B, Qiao M, Lu F (2012). Composition, nutrition, and utilization of okara (soybean residue). Food Rev Int.

[CR38] Lin YL, Wang TH, Lee MH, Su NW (2008). Biologically active components and nutraceuticals in the *Monascus*-fermented rice: a review. Appl Microbiol Biotechnol.

[CR39] Li-Ning PU, Guangjing C, Jianquan K (2017). Optimization of fermentation process of *Monascus* barley bran and coix seed by response surface methodology. Food Sci.

[CR40] Maloney K, Truong VD, Allen JC (2014). Susceptibility of sweet potato (*Ipomoea batatas*) peel proteins to digestive enzymes. Food Sci Nutr.

[CR41] Manan MA, Webb C (2019). Insights into physical characterization of solid state fermentation: from preliminary knowledge to practical application. J Biotech Res.

[CR42] Miyake T, Kono I, Nozaki N, Sammoto H (2008). Analysis of pigment compositions in various *Monascus* cultures. Food Sci Technol Res.

[CR43] Moorthy M, Viswanathan K (2009). Nutritive value of extracted coconut (*Cocos nucifera*) meal. Res J Agric Biol Sci.

[CR44] Mousa SA, Abdou DAM, Mohamed GA, Abo-El-Seoud MA, Eldin AAK, El-mehalawy AA (2018). Production of red pigment by *Monascus purpureus* NRRL 1992 under submerged and solid-state fermentation. Egypt J Microbiol.

[CR45] Nimnoi P, Lumyong S (2011). Improving solid-state fermentation of *Monascus purpureus* on agricultural products for pigment production. Food Bioprocess Technol.

[CR46] Nugerahani I, Sutedja AM, Srianta I, Widharna RM, Marsono Y (2017). In vivo evaluation of *Monascus*-fermented durian seed for antidiabetic and antihypercholesterol agent. Food Res.

[CR47] Okpako CE, Ntui V, Osuagwu AN, Obasi FI (2008). Proximate composition and cyanide content of cassava peels fermented with *Aspergillus niger* and *Lactobacillus rhamnosus*. J Food Agric Environ.

[CR48] Padmavathi T, Prabhudessai T (2013). A solid liquid state culture method to stimulate *Monascus* pigments by intervention of different substrates. Int Res J Biol Sci.

[CR49] Patakova P (2013). *Monascus* secondary metabolites: production and biological activity. J Ind Microbiol Biotechnol.

[CR50] Pattanagul P, Pinthong R, Phianmongkhol A, Leksawasdi N (2007). Review of angkak production (*Monascus purpureus*). Chiang Mai J Sci.

[CR51] Prayoga MJ, Tjiptaningrum A (2016). Pengaruh pemberian angkak (beras fermentasi *Monascus purpureus*) dalam meningkatkan kadar trombosit pada penderita demam berdarah dengue (The effect of giving angkak (*Monascus purpureus* fermented rice) in increasing platelet levels in patients with dengue fever). Med J Lampung Univ.

[CR52] Rajeswari TR, Ponnusami V, Sugumaran KR (2014). Production of *Monascus* pin low cost fermentation. IJCRGG.

[CR53] Razak DLA, Rashid NYA, Jamaluddin A, Sharifudin SA, Long K (2015). Enhancement of phenolic acid content and antioxidant activity of rice bran fermented with *Rhizopus oligosporus* and *Monascus purpureus*. Biocat Agric Biotechnol.

[CR54] Sehrawat E, Panesar PS, Panesar R, Kumar A (2017). Biopigment produced by *Monascus purpureus* MTCC 369 in submerged and solid state fermentation: a comparative study. Pigm Resin Technol.

[CR55] Sehrawat R, Panesar PS, Swer TL, Kumar A (2017). Response surface methodology (RSM) mediated interaction of media concentration and process parameters for the pigment production by *Monascus purpureus* MTCC 369 under solid state fermentation. Pigm Resin Technol.

[CR56] Sepelev I, Galoburda R (2015) Industrial potato peels waste application in food production: a review. In: Research for Rural Development. International Scientific Conference Proceedings (Latvia). Latvia University of Agriculture. 1, 130–136, 2015

[CR57] Shao Y, Lei M, Mao Z, Zhou Y, Chen F (2014). Insights into *Monascus* biology at the genetic level. Appl Microbiol Biotechnol.

[CR58] Shi YC, Pan TM (2012). Red mold, diabetes, and oxidative stress: a review. Appl Microbiol Biotechnol.

[CR59] Srianta I, Harijono H (2015). *Monascus*-fermented sorghum: pigments and monacolin K produced by *Monascus purpureus* on whole grain, dehulled grain and bran substrates. Int Food Res J.

[CR60] Srianta I, Hendrawan B, Kusumawati N, Blanc PJ (2012). Study on durian seed as a new substrate for angkak production. Int Food Res J.

[CR61] Srianta I, Kusumawati N, Nugerahani I, Artanti N, Xu GR (2013). In vitro α-glucosidase inhibitory activity of *Monascus*-fermented durian seed extracts. Int Food Res J.

[CR62] Srianta I, Nugerahani I, Kusumawati N, Subianto C, Tewfik S, Tewfik I (2014). Therapeutic antioxidant activity of *Monascus*-fermented durian seed: a potential functional food ingredient. IJFNPH.

[CR63] Srianta I, Ristiarini S, Nugerahani I, Sen SK, Zhang BB, Xu GR, Blanc PJ (2014). Recent research and development of *Monascus* fermentation products. Int Food Res J.

[CR64] Srianta I, Zubaidah E, Estiasih T, Yamada M, Harijono,  (2016). Comparison of *Monascus purpureus* growth, pigment production and composition on different cereal substrates with solid state fermentation. Biocat Agric Biotechnol.

[CR65] Srianta I, Zubaidah E, Estiasih T, Iuchi Y, Harijono YM (2017). Antioxidant activity of pigments derived from *Monascus purpureus*-fermented rice, corn, and sorghum. Int Food Res J.

[CR66] Statista (2020) Grain production worldwide 2018/19, by type. https://www.statista.com/statistics/263977/world-grain-production-by-type/. Accessed 21 June 2020

[CR67] Su Y, Wang J, Lin T, Pan T (2003). Production of the secondary metabolites gamma-aminobutyric acid and monacolin K by *Monascus*. J Ind Microbiol Biotechnol.

[CR68] Sun C, Wu X, Chen X, Li X, Zheng Z, Jiang S (2020). Production and characterization of okara dietary fiber produced by fermentation with *Monascus anka*. Food Chem.

[CR69] Tan J, Chu J, Wang Y, Zhuang Y, Zhang S (2014). High throughput system for screening of *Monascus purpureus* high-yield strain in pigment production. Bioresour Bioprocess.

[CR70] Velmurugan P, Hur H, Balachandar V, Kamala-Kannan S, Lee KJ, Lee SM, Chae JC, Shea PJ, Oh BT (2011). *Monascus* pigment production by solid-state fermentation with corn cob substrate. J Biosci Bioeng.

[CR71] Vendruscolo F, Tosin I, Giachini AJ, Schmidell W, Ninow JL (2014). Antimicrobial activity of *monascus* pigments produced in submerged fermentation. J Food Process Preserv.

[CR72] Venkateswaran V, Vijayalakshmi G (2010). Finger millet (*Eleusine coracana*)—an economically viable source for antihypercholesterolemic metabolites production by *Monascus purpureus*. J Food Sci Technol.

[CR73] Watanabe A, Ebizuka Y (2004). Unprecedented mechanism for chain length determination in fungal aromatic polyketide synthases. Chem Biol.

[CR74] Wen Q, Cao X, Chen Z, Xiong Z, Liu J, Cheng Z, Zheng Z, Long C, Zheng B, Huang Z (2020). An overview of *Monascus* fermentation processes for monacolin K production. Open Chem.

[CR75] Yang Y, Liu B, Du X, Li P, Liang B, Cheng X, Du LC, Huang D, Wang L, Wang S (2015). Complete genome sequence and transcriptomics analyses reveal pigment biosynthesis and regulatory mechanismsin an industrial strain, *Monascus purpureus* YY-1. Sci Rep.

[CR76] Zubaidah E, Dewi AP (2014). Effect addition of rice bran on fermentation process to increasing lovastatin and intensity of red pigment angkak. Adv J Food Sci Technol.

